# Deep Vein Thrombosis Is Facilitated by Endothelial-Derived Extracellular Vesicles via the PDI–GRP94–GPIIb/IIIa Pathway in Mice

**DOI:** 10.3390/jcm12134265

**Published:** 2023-06-26

**Authors:** Hongtao Lan, Zhoujie Tong, Yaqiong Jiao, Haitao Han, Ying Ma, Yulin Li, Xu Jia, Boang Hu, Wei Zhang, Ming Zhong, Zhihao Wang

**Affiliations:** 1 The Key Laboratory of Cardiovascular Remodeling and Function Research, Chinese Ministry of Education, The State and Shandong Province Joint Key Laboratory of Translational Cardiovascular Medicine, Department of Cardiology, Cheeloo College of Medicine, Qilu Hospital, Shandong University, Chinese National Health Commission and Chinese Academy of Medical Sciences, Jinan 250012, China; lanxiaofei755@163.com (H.L.);; 2 Department of Geriatric Medicine, Cheeloo College of Medicine, Qilu Hospital, Shandong University, Jinan 250012, China; 3 Department of General Practice, Cheeloo College of Medicine, Qilu Hospital, Shandong University, Jinan 250012, China

**Keywords:** platelet, endothelial-derived extracellular vesicles, deep vein thrombosis, protein disulfide isomerase

## Abstract

Aims: Deep vein thrombosis (DVT) is a prevalent cardiovascular condition. Endothelial-derived extracellular vesicles (EVs) may play a crucial role in platelet-dependent DVT development via platelet activation, but the mechanism is not clear yet. This research aims to understand how platelets and endothelial-derived EVs work in DVT. Methods: The interaction between protein disulfide isomerase (PDI) and glucose-regulated protein 94 (GRP94) was founded by molecular docking. Inferior vena cava stasis–induced mice received PDI and GRP94 inhibitor treatments. Platelet activation, endothelial-derived EVs, and PDI were measured using flow cytometry. The expression of PDI and dimetric GRP94 in platelets co-cultured with hypoxic endothelial cells was confirmed by Western blot or native PAGE. The fluorescence resonance energy transfer assay shows conformational changes in GPIIb/IIIa on platelet surfaces. A tracking experiment was performed using PKH26, which labelled endothelial-derived EVs, and the endocytosis of EVs by platelets was tracked by confocal microscope. Results: In a DVT mouse model, platelets enhance venous thrombus formation in a coagulation-independent manner, instead, platelet activation and the length of the thrombus are related to PDI and GRP94 activity. Next, we found that the expression level of endothelial-derived EVs carrying PDI is significantly increased in plasma. Endothelial-derived EVs carrying PDI are endocytosed by platelets, in which the content of GRP94 dimer is elevated, and consequently increases the expression of surface GPIIb/IIIa. In addition, PDI allosterically interacts with GPIIb/IIIa, which is re-configurated into an activated form. Conclusion: Endothelial-derived EVs carrying PDI induce DVT via interplay with GRP94 and GPIIb/IIIa in platelets. These findings emphasize the significance of platelets in DVT formation, and PDI may be a suitable target in DVT prevention.

## 1. Introduction

Deep vein thrombosis (DVT) and pulmonary embolism are called venous thromboembolism (VTE), which is a very costly health problem worldwide [1]. In China, the hospitalization rate for VTE rose by more than five times between 2007 and 2016 [2]. Understanding the mechanisms could have a significant impact on the development of novel and safe DVT therapeutic strategies.

Endothelial-derived extracellular vesicles (EVs), a potent pro-thrombotic agent carrying a variety of signals that initiate platelet activation, are released from the damaged venous endothelium barrier. Holbrook’s research indicates that the procoagulant effect of platelet-derived EVs is 50–100 times higher than that of activated platelets, and platelet-derived microparticles have an amplification effect on procoagulant cascade [3]. It is reported that elevated plasma levels of platelet-derived EVs are associated with future risk of VTE incident, which confirms the role of platelet activation in the pathogenesis of VTE [4].

Configurational changes of GPIIb/IIIa receptors are involved in the formation of thrombi in pathologic condition [5]. Using specific single-chain antibodies to target and antagonize the active conformation of GPIIb/IIIa could efficiently inhibit thrombosis without increasing the bleeding time [6]. Protein disulfide isomerase (PDI) is shown to control GPIIIa component isomerization via thiol–disulfide bond modification [7], thereby altering the steric structure of GPIIb/IIIa, and the activated GPIIb/IIIa receptor bears increased potential for platelet aggregation and fibrinogen cross-linking [8]. Phase II clinical trials show that the PDI inhibitor isoquercetin reduces the levels of D-dimer and soluble P-selectin in patients with advanced cancer, and no patient was reported to have DVT (NCT02195232) [6]. Preclinical studies demonstrate that platelet-derived PDI has an important role in mediating platelet accumulation but not in the initial phases of platelet adhesion, therefore, it is not required for normal hemostasis [9]. Interestingly, PDI released from damaged endothelial cells seems to mediate fibrinogen generation and thrombus formation in vivo [10]. However, the precise mechanisms involved in the antithrombotic effects of PDI remain to be elucidated. We hypothesize that PDI on endothelial-derived EVs could regulate the activation of platelet GPIIb/IIIa receptors without affecting the normal hemostatic function.

Glucose regulatory protein 94 (GRP94) is an analog of the heat shock protein located in the endoplasmic reticulum. It is involved in the conformational regulation of secreted proteins and has been confirmed to play an important role in the signal transduction and activation of GPIIb/IIIa receptors [11]. A GRP94 inhibitor, Kung65, can significantly reduce the expression of platelet surface GPIIb/IIIa receptors [11]. However, the currently used GRP94 inhibitors are mostly N-terminal binding inhibitors, which can lead to a series of heat shock responses and therapeutic toxicity. Monomeric GRP94 is connected through a C-terminal disulfide bond and composed into a dimer to attain biological function [12]. Intriguingly, both PDI and GRP94 are highly conserved proteins located in the endoplasmic reticulum, and there are some overlaps in their distribution and expression [13]. PDI regulates protein functions by mediating the structure of disulfide bonds. The effects of PDI-induced changes of monomers into dimers on GRP94 remain unknown.

In the present study, in DVT model mice, we firstly found that elevated endothelial-derived EVs carrying PDI released by injured venous endothelium could regulate the steric conformation of platelet surface GPIIb/IIIa. Furthermore, PDI could increase the biological activity of GRP94 by altering the conformation of disulfide bonds, causing an increased expression of platelet surface GPIIb/IIIa. These findings may pave the way for the clinical application of PDI in the prevention of DVT.

## 2. Materials and Methods

All animal procedures were approved by the Ethics Committee of Qilu Hospital of Shandong University. This study was conducted in full adherence to relevant regulations and guidelines. 

### 2.1. Animal Model 

At first, eight-week-old C57BL/6 mice (male, in order to exclude the sex hormones factor) were purchased from SPF Biotechnology Co., Ltd. (Beijing, China) and kept in the animal room of the cardiovascular laboratory at Qilu Hospital of Shandong University. CCF642 (a PDI inhibitor; MedChemExpress, Monmouth Junction, NJ, USA) and GRP94 inhibitor-1 (a GRP94 inhibitor; MedChemExpress) were purchased. GRP94 inhibitor-1 inhibits GRP94 expression in the endoplasmic reticulum [14]. CCF642 has been used in mice experiments [15]. Mice were randomized into four groups: sham operation group (mice underwent the same surgical procedure without IVC stenosis and have no treatment, n = 7), DVT group (mice underwent the IVC stenosis operation and were injected with 200 μL of saline via intraperitoneal injection, n = 8), DVT and PDI inhibitor group (mice underwent the IVC stenosis operation and were injected with 5 mg/kg of CCF642 diluted in 200 μL of saline via intraperitoneal injection, n = 8), and DVT and GRP94 inhibitor group (mice underwent the IVC stenosis operation and were injected with 15 mg/kg of GRP94 inhibitor-1 diluted in 200 μL of saline via intraperitoneal injection, n = 7). The presented sample sizes of the different experimental groups include only the mice that survived after treatment and the DVT model operation. The time of CCF642 treatment was 3 times a week for 3 weeks. The time of GRP94 inhibitor-1 treatment was 10 consecutive days.

Due to the nationwide epidemic, isoflurane or ketamine could not be purchased in the province. Since we performed platelet-related experiments, we chose tribromoethanol (intra-peritoneal 300 mg/kg in 0.9% NaCl) as an anesthetic, which has a low hemodynamic impact. We also strictly controlled the anesthesia time and observed no adverse reactions in mice. Upon attaining an appropriate plane of anesthesia, the DVT model was established after treatment on the 21st day. The construction of the DVT model was referred to as the inferior vena cava (IVC) flow restriction model. In brief, mice were anesthetized and placed in a supine position. The abdomen was opened along the midline, and the intestines were exteriorized gently. Warmed saline was applied to the intestine during the surgery to avoid dehydration. The IVC was gently separated the from the aorta and then ligated under the left renal vein with a 7–0 polypropylene suture over a spacer (30-gauge needle). After that, the spacer was removed to create a 90% stenosis without endothelial disruption. All visible side branches were ligated completely. At last, the intestines were returned to the peritoneal cavity. Peritoneum and skin were closed with 6–0 sutures. Mice were sacrificed at 48 h after the operation, and their retro-orbital venous blood and thrombi were obtained. A similar procedure without IVC ligation was performed on mice in the sham operation group.

Mice were euthanized using tribromoethanol anesthesia (intraperitoneal 300 mg/kg in 0.9% NaCl), followed by exsanguination for the blood collection.

### 2.2. Isolation of Platelets and Plasma

All blood samples were collected in EP tubes containing 0.109 mol/L sodium citrate at room temperature. The blood samples were centrifuged at 800 rpm for 8 min and platelet-rich plasma (PRP) was obtained from supernatant. The platelet-rich plasma was centrifuged at 2300 rpm for 10 min to precipitate platelets. Platelet-poor plasma (PPP) was preserved at −80 °C. Platelets were re-suspended and the concentration was adjusted to 1 × 10^6^ cells/mL for later use. All procedures were performed gently, and fresh platelets was immediately used for experiment to avoid undesirable activation of platelets.

### 2.3. Clot Retraction

Briefly, PRP (500 μL) was poured into a polyethylene tube and samples were pre-incubated in different handles for 15 min at 37 °C, and clot retraction was triggered by adding thrombin. The samples were re-suspended in human fibrinogen (400 μg/mL; Sigma, Kawasaki, Japan). Clot retraction was triggered by adding thrombin (1 U/mL; Solabio, Beijing, China) at 37 °C. Clot retraction was monitored every 5 min and photographed. Clot size was quantified from photographs using Image J v1.8.0.112 software.

### 2.4. Platelet Spreading on Immobilized Fibrinogen

Chamber slides with microtiter wells were coated with 10 μg/mL fibrinogen in 0.1 M NaHCO_3_ (pH 8.3) at 4 °C overnight. Washed platelets (2 × 10^7^/mL) were allowed to adhere to and spread on fibrinogen-coated wells at 37 °C for 2 h with stimulation by thrombin. After washing, the cells were fixed, permeabilized, and stained with Alexa Fluor 488-conjugated phalloidin. Images were acquired and the spreading area of single platelets was measured using Image J software.

### 2.5. Flow Cytometry (FCM)

CD144 has been proposed as one of the most specific markers for the detection of endothelial-derived EVs because it has not yet been found to be expressed on any other blood cell in humans [16]. GPIIb/IIIa is a platelet activation marker [17]. GPIIIa (CD61) integrin is a subunit of GPIIb/IIIa; when expressed on platelets, it is the most abundant glycoprotein on the platelet membrane, and it is also the platelet fibrinogen receptor that associates with CD41 [18]. GPIIb (CD41) integrin is a subunit of GPIIb/IIIa and is expressed on the platelet surface [19]. P-selectin (CD62p) is a platelet activation marker [20]. The presence of PDI in EVs was detected through dichromatic FCM of CD144 and PDI. A total of 20 μL of PPP was added into a BD tube, followed by 2 μL of anti-mouse CD144 antibody (eBioscience, San Diego, CA, USA) and 2 μL of anti-mouse PDI antibody (Santa Cruzm, Dallas, TX, USA). Fluoresbrite^®^ microparticles (Polysciences, Warrington, PA, USA) were premixed with phosphate-buffered saline (PBS) in 1 to 1000 volumes. Antibodies and Fluoresbrite^®^ microparticles were mixed to obtain a final volume of 100 μL. The mixture was incubated at 37 °C for 20 min in the dark. Finally, 200 μL 4% paraformaldehyde was added. The samples were immediately subjected with FACS Aria.

Platelet activation markers in mice platelets were detected by dichromatic FCM. A total of 20 μL of platelet suspension was placed in a BD FCM test tube, along with 3 μL of CD62p (Biolegend, San Diego, CA, USA) and 2 μL of CD61 (Biolegend) or 2 μL of CD41 (Biolegend); PBS was added to obtain a total volume of 100 μL. The mixture was gently mixed and incubated at 37 °C for 20 min in the dark. Subsequently, 200 μL of 4% paraformaldehyde was added, and the samples were immediately subjected to FACS Calibe. Platelet activation markers in human-derived platelets were detected by the above procedure. PAC-1 (Biolegend) is a marker for activated GPIIb/IIIa on the human platelet surface. FCM data were analyzed with FlowJo v10 software.

### 2.6. Histological Examination

The thrombi, together with the surrounding venous walls, were harvested on the 2nd day after the operation. The specimens along the whole thrombus were fixed with 4% paraformaldehyde, embedded in paraffin, and sliced into 5 μm sections. HE staining, immunohistochemistry (IHC) staining, and immunofluorescent (IF) staining were conducted according to provided instructions. Antibody against CD41 (Abcam, Cambridge, UK) and antibody against fibrinogen (Abcam) were used in IHC. Antibodies against GRP94 (Abcam) and CD41 were applied to IF. The thrombolytic ratio was referred to as the specific value of the distance between the thrombus and the endothelium with the diameter of venous lumen [21]. Images from 5 to 8 randomly selected visual fields (magnification, ×400) of each sample were captured, and the positive-staining area was measured by using Image-Pro Plus V7.0 software and compared among groups.

### 2.7. Cell Culture

The 293T cells were purchased from Zhong Qiao Xin Zhou (Shanghai, China). Primary human umbilical vein endothelial cells (HUVECs) were purchased from ScienCell company (Carlsbad, CA, USA) and identified by CD31 (Abcam, Supplementary Figure S1). HUVECs were incubated at 37 °C in a humidified atmosphere with 5% CO_2_. Endothelial cell medium supplemented with 5% fetal bovine serum and penicillin/streptomycin (final concentration 100 IU/mL) was purchased from the ScienCell company. The hypoxia injury model of HUVEC was performed under 1% O_2_, 5% CO_2_, and 94% N_2_ in an acrylic chamber; O_2_ was maintained using a CO_2_ and O_2_ controller.

### 2.8. Migration Assay

HUVECs were seeded in six-well cell culture plates and allowed to reach 80% confluence, and then the monolayer culture was scratched using sterile 200 μL pipette tips. The culture medium was changed to remove any floating cells. The fresh culture medium did not contain FBS. Microphotographs of wound closure were taken at 0 h, 6 h, 12 h, and 24 h after cell wounding (Supplementary Figure S2). The locations of the microphotographs were marked on the plates. The percentage of gap remaining was also quantitatively calculated.

### 2.9. Extracellular Vesicles (EVs) Extraction

When the density of HUVECs reached 70% to 80% in the field, the culture medium was refreshed with ECM containing 5% microparticle-depleted fetal bovine serum and cultured for 24 h. Thereafter, supernatants from cultured HUVECs were centrifuged at 3000× *g* for 10 min to remove cellular debris. Cell supernatants and the concentration EVs solution were mixed and incubated for 2 h at 4 °C. Subsequently, the mixture was centrifugated at 1000× *g* for 1 h at 4 °C, and the supernatants were gently removed. The sediment was resuspended and filtered through an EV purification filter, and then it was centrifuged at 3000× *g* for 10 min at 4 °C. The EVs were identified by Western blot (Supplementary Figure S3). The EVs were resuspended in PBS and stored at −80 °C for further use.

### 2.10. PKH26 Staining

HUVECs were suspended, and the cell density was adjusted to 1 × 10^7^ cells/mL. A total of 2 mL of cell suspension was centrifuged at 400× *g* for 5 min. The supernatant was removed gently and the cell pellet was resuspended by 1 mL of an aqueous solution. A total of 4 μL of PKH26 ethanolic dye solution was diluted into another 1 mL of an aqueous solution. The cell suspension and dye solution were immediately mixed and incubated for 5 min. The staining was terminated by adding 2 mL of FBS, and then the cells were washed by complete medium 3 times. Thereafter, the cell pellet was resuspended in 10 mL of EVs-free complete medium and cultured. EVs labeled by PKH26 were extracted as previously described. EVs were incubated with fresh platelets for 2 h and immediately subjected to a fluorescence confocal microscope (Zeiss LSM710, GER).

### 2.11. Fluorescence Resonance Energy Transfer (FRET)

EGFP-labeled GPIIb overexpression vector was purchased from Biosune (Shanghai, China). mCherry-labeled GPIIIa overexpression vector (Sp-mcherry-NM_000212) was purchased from Biosune (Shanghai, China). All primer information and sequences are shown in Supplementary Table S1.

The 293T cells were cultured in a 15 mm glass-bottomed cell culture dish with a cell density of over 50%. Each vessel was transfected by 1 μg of reconstructed vectors mixed with 2 μL of P3000. A total of 2 μL of lipofectamine 3000 transfection reagent (Invitrogen, Carlsbad, CA, USA) was premixed with vectors in 600 μL of optimen (Invitrogen) to facilitate the transfection. Optimen was replaced by a regular medium 4 h after transfection. Transfected cells were cultured for another 48 h, and extracted endothelial-derived EVs and PDI inhibitor were incubated for 2 h. Thereafter, 293T cells were subjected to a laser scanning confocal microscope.

### 2.12. Platelet Aggregometry

Human platelets were resuspended to 6 × 10^8^ cells/mL in Tyrode’s solution. Aggregation was triggered by Adenosine 5′-diphosphate (ADP; GLPBIO, Montclair, CA, USA). Reactions were performed by stirring ×1200 rpm at 37 °C in a Chronolog four-channel optical aggregation system (Chrono-log, Havertown, PA, USA). A blank control of Tyrode’s solution was used as a contrast. The absorbances of the control group and the experimental group were calibrated to the same level at first, and ADP was immediately introduced to the tested channels. The stirring was prolonged for 5 min until the absorbance curve was flattened and the aggregation rate was exported for analysis.

### 2.13. Western Blot

Proteins were extracted from platelets or endothelial-derived EVs, separated on 10–12% sodium dodecyl sulfate–polyacrylamide gels, and transferred onto PVDF membranes, which were soaked in Tris-buffered saline–Tween (TBST) solution containing 5% bovine serum albumin (room temperature) to block non-specific binding. Subsequently, the membranes were exposed to the primary antibodies: anti-PDI (Abcam), anti-ERP57 (Abcam), anti-ERP46 (Immunoway, Plano, TX, USA), anti-GRP94 (Abcam), anti-CD63 (Abcam), anti-Calnexin (Abcam), and anti-TSG101 (Abcam). After staining overnight at 4 °C, the membranes were washed three times with TBST and incubated with horseradish peroxidase (HRP)-labeled anti-rabbit (ZSGB-BIO, Beijing, China) or anti-mouse (ZSGB-BIO) secondary antibodies at room temperature for 90 min. After washing thrice more with TBST, an enhanced chemiluminescence (ECL) reagent was added, and images were acquired and analyzed quantitatively using Image J.

### 2.14. Statistical Analysis

At least three biological replicates of each experiment were performed. Data are expressed as the mean ± SEM. The Shapiro–Wilk test was conducted to evaluate normality. A one-way ANOVA and the Kruskal–Wallis test were performed for parametric and non-parametric data, respectively. SPSS 26.0 was used for data analysis. The following *p* values were considered significant: * *p* < 0.05, ** *p* < 0.01, *** *p* < 0.001.

## 3. Results

### 3.1. DVT Mouse Model Establishment and Platelet Activation in Thrombus Formation

**Figure 1:**
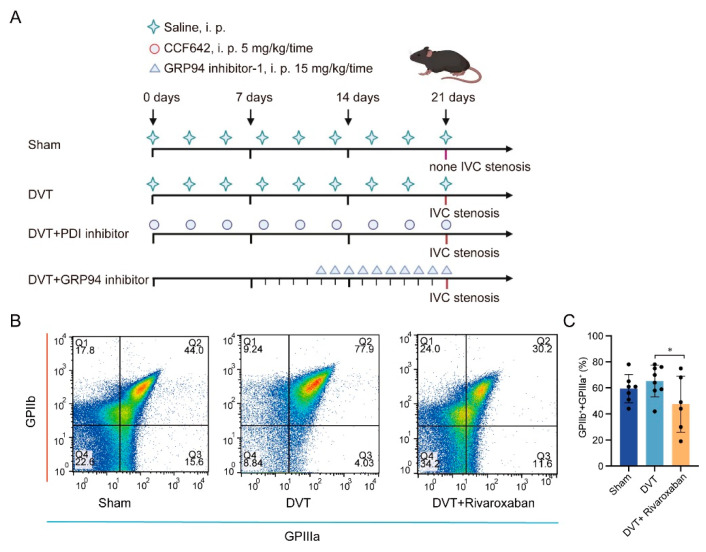
Platelet activation in thrombus formation. (**A**) Scheme of systemic injection. The time of saline injection is 21 consecutive days from day 1 in sham group and DVT group. The time of CCF642 treatment is 3 times a week for 3 weeks from day 1. The time of GRP94 inhibitor-1 treatment was 10 consecutive days from day 12. (**B**) Detection of CD62p on the surface of platelets from mice by FCM. (**C**) Statistical analysis of GPII/IIIa percentage on platelets. * *p* < 0.05. DVT, deep vein thrombosis.

As shown in Figure 1A, nine-week-old mice were selected randomly and divided into four groups: the sham group (PBS), the DVT group (PBS), the DVT and PDI inhibitor group (5 mg/kg/day, three times a week for 3 weeks), and the DVT and GRP94 inhibitor (15 mg/kg/day, 10 consecutive days). Mice were operated on after treatment on the 21st day, and the process of the DVT model is shown in Supplementary Figure S4A,B. The survival and incidence of the DVT model were evaluated. No mice died in the sham group, and two mice died in the DVT group (lateral branches of the inferior vena cava were torn accidentally, which led to death). No thrombi were found in the sham group. In the DVT group, the incidence of DVT reached 63%, and the length of the thrombi was 3.7 ± 3.6 mm. In the sham group, no abnormalities, such as thrombus formation, were observed. The thrombi were adherent to the vessel wall. The color of the thrombi was black, and their attribute was tough (Supplementary Figure S4C).

HE staining shows that the normal mouse inferior vena cava tissue structure is intact and undamaged. After the DVT operation, a large amount of focal inflammation and exudation of the vessel wall and interstitial tissue are seen; the vessel wall becomes thinner, and the thrombi extends to the vessel lumen (Supplementary Figure S4D). In summary, the mouse DVT model is successfully established.

Seeking to examine the specific role of platelets in DVT, it is found by IHC assay that platelet activation marker GPIIb/IIIa occupies a large proportion of the thrombus surface (Supplementary Figure S4E). It is shown by the ELISA assay results that P-selectin is significantly higher in the peripheral blood of mice in the DVT group compared with the sham group (*p* < 0.001, Supplementary Figure S4F). The expression level of CD62p platelets is significantly higher in the DVT group compared with the sham group, as assessed by FCM (*p* < 0.05, Supplementary Figure S4G). The above results imply that activated platelets might be involved in DVT.

For the DVT and rivaroxaban group (mice underwent the IVC stenosis operation and were injected with 3.0 mg/kg of rivaroxaban diluted in 200 μL of saline via intraperitoneal injection, n = 6), a direct FXa inhibitor was used as a positive control for coagulation function. Interestingly, FCM reveals that the GPIIb/IIIa level on the surface of the activated platelets is significantly lower in the DVT and rivaroxaban group compared with the DVT group (Figure 1B,C). These observations are further evidence that activated platelets are ideal receptors for targeting thrombi, since aggregation of activated platelets mediated by fibrinogens is a significant hallmark event in DVT. Taken together, these data reflect the fact that the DVT model was successfully established, and they reflect the critical importance of platelet activation in DVT.

### 3.2. PDI and GRP94 Accelerated Thrombus Formation Mediated by Increased Platelet Activation Levels in Plasma

**Figure 2:**
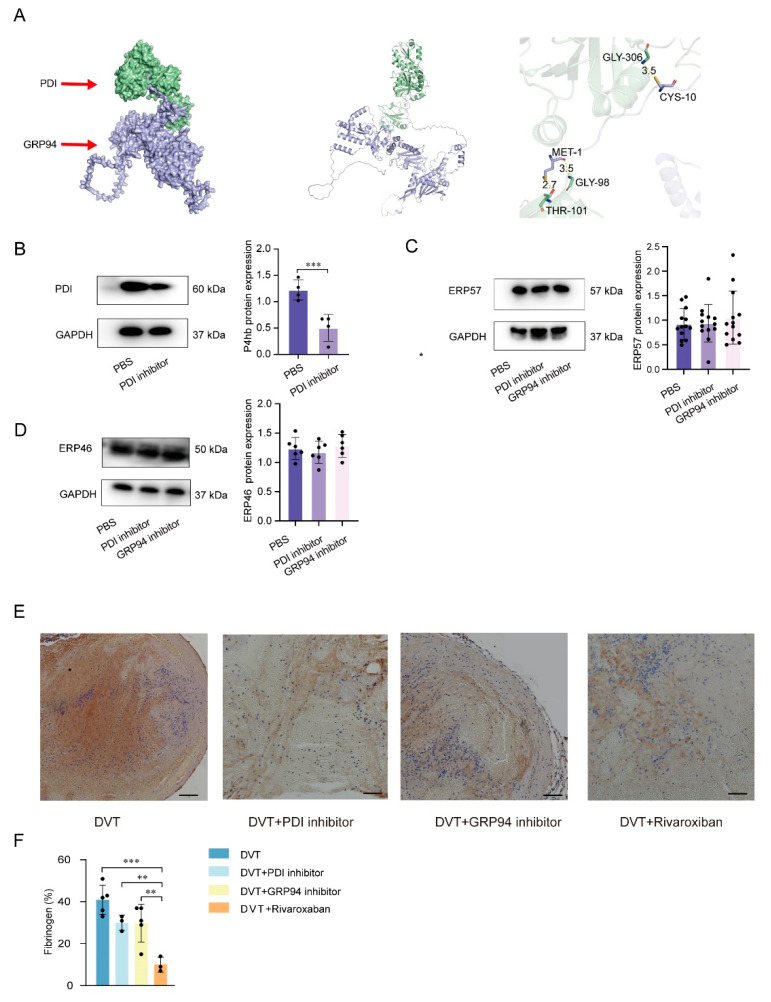
PDI and GRP94 accelerated thrombus formation mediated by increased platelet activation levels in plasma. (**A**) The docking results show that PDI and GRP94 could form a protein–protein complex with a high Z-dock docking fraction (1608.029). PDI interacts with the GRP94 protein mainly through the formation of hydrogen bonds as well as hydrophobic interactions. MET-1 and CYS-10 of GRP94 form hydrogen bonds with THR-101, GLY-98, and GLY-306 of PDI. CYS-10 is associated with disulfide bond formation. (**B**) Human platelets were co-cultured with PDI inhibitor or PBS-negative control to detect PDI inhibitor specificity; figure shows representative Western blot bands of PDI in platelets. (**C**) Human platelets were stimulated by PBS negative control, PDI inhibitor, and GRP94 inhibitor; figure shows representative Western blot bands of ERP57 in platelets. (**D**) Human platelets were stimulated by PBS-negative control, PDI inhibitor, and GRP94 inhibitor; figure shows representative Western blot bands of ERP46 in platelets. (**E**) Representative microscope images displaying fibrinogen on thrombi of mice by immunohistochemical staining. Scale bar = 100 μm. (**F**) Quantitative analysis of fibrinogen as a percentage of total area on thrombi. * *p* < 0.05, ** *p* < 0.01, *** *p* < 0.001. PBS, phosphate buffer saline; DVT, deep vein thrombosis; PDI, protein disulfide isomerase; GRP94, glucose-regulated protein 94.

To determine whether there had been an interaction between PDI and GRP94, we used molecular docking, which indicated that the PDI target was GRP94 (Figure 2A). A PDI inhibitor and a GRP94 inhibitor were used to detect the effects of PDI and GRP94 on platelet activation in the DVT mice model. The PDI content in platelets is significantly lower in the PDI inhibitor group than in the PBS group (Figure 2B), as determined by Western blot. We further detect endoplasmic reticulum stress indicators ERP57 and ERP46. Western blot analysis reveals that ERP57 and ERP46 contents are not significantly different in the PDI inhibitor group and the GRP94 inhibitor group when compared to PBS group (Figure 2C,D). These results suggest that the PDI inhibitor shows remarkable selectivity for PDI. AST, ALT, and LDH, and CK shows no significant changes between the PDI inhibitor group and the GRP94 inhibitor group in the peripheral blood of mice, as determined by ELISA (*p* > 0.05, Supplementary Figure S5A–D). These results suggest that the PDI inhibitor or the GRP94 inhibitor could specifically act on PDI or GRP94 and not cause dysfunctions of the organs, such as liver and heart damage.

Both the PDI inhibitor and the GRP94 inhibitor decrease the length of the thrombi (*p* < 0.01, Table 1). Furthermore, it is shown by HE staining assay that the thrombi fills the entire vessel wall in different groups (Supplementary Figure S5E). The above results indicate that the PDI inhibitor and the GRP94 inhibitor attenuate thrombus formation.

In the mouse model of DVT, PDI inhibitor or GRP94 inhibitor injection was performed. Mice that received treatment show the characteristics of lower P-selectin levels and decreased vWF levels (both *p* < 0.05, Supplementary Figure S5F). Meanwhile, the PDI inhibitor and the GRP94 inhibitor reduce fibrinogen levels to levels similar to those seen in the sham group, so we excluded the influence of coagulation factors on DVT (Supplementary Figure S5G). Furthermore, we detect fibrinogen on the surface of the thrombi with IHC, and the fibrinogen expression level is similar in the DVT and PDI inhibitor group and the DVT and GRP94 inhibitor group when compared to DVT group (Figure 2E,F). In other words, the knockdown of PDI or GRP94 obviously attenuates thrombi formation but does not depend on coagulation factors.

### 3.3. Inhibition of PDI and GRP94 Represses the GPIIb Submit, the GPIIIa Submit, and the GPIIb/IIIa Integrin Expression upon Platelet Activation

**Figure 3:**
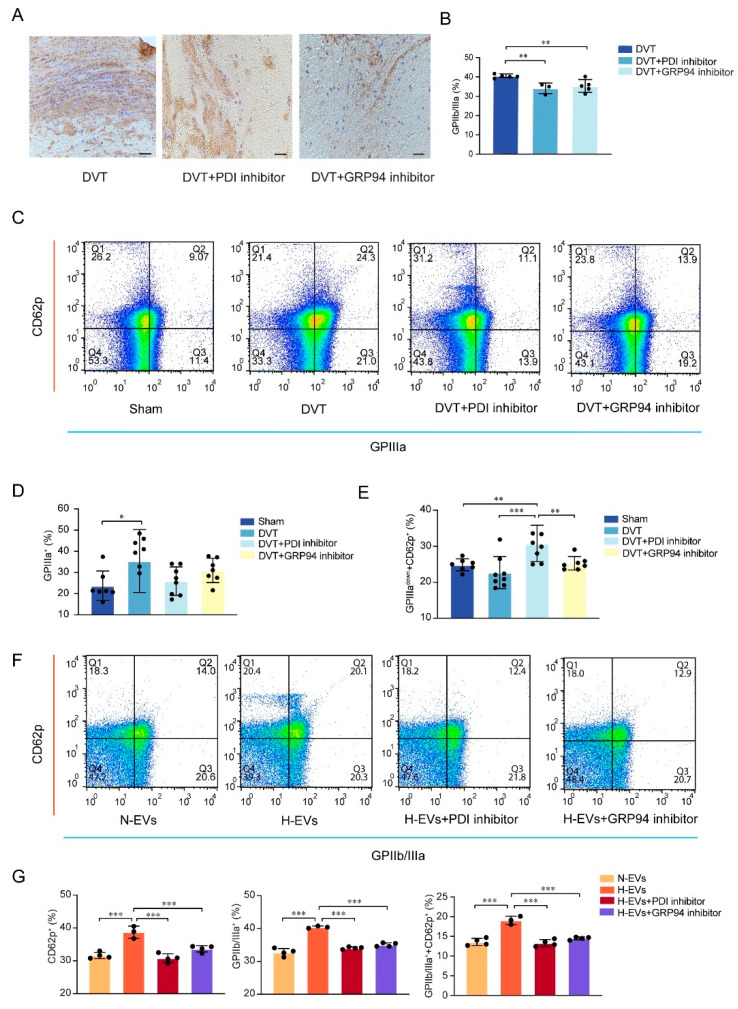
Inhibition of PDI and GRP94 represses the GPIIb submit, the GPIIIa submit, and the GPIIb/IIIa integrin expression upon platelet activation. (**A**) Representative microscope images displaying GPIIb/IIIa on thrombi from mice by immunohistochemical staining. Scale bar = 40 μm. (**B**) Quantitative analysis of GPIIb/IIIa as a percentage of total area on thrombi. (**C**) Platelets were extracted from peripheral blood in mice, and the expressions of CD62p and the GPIIIa subunit on platelet surfaces was detected by FCM. (**D**) Statistical analysis of the GPIIIa subunit percentage on platelets. (**E**) Statistical analysis of the GPIIIa subunit down percentage in CD62p platelets. (**F**) Platelets and endothelial-derived EVs were co-cultured. The expression of CD62p and GPIIb/IIIa integrin on platelet surfaces was detected by FCM. (**G**) Statistical analysis of CD62p and GPIIb/IIIa integrin percentage on platelets. EVs, extracellular vesicles; * *p* < 0.05, ** *p* < 0.01, *** *p* < 0.001. DVT, deep vein thrombosis; PDI, protein disulfide isomerase; GRP94, glucose-regulated protein 94.

We further investigated the underlying mechanisms of platelet activation in DVT. IHC analysis reveals that GPIIb/IIIa is significantly reduced on thrombus tissue in the DVT and PDI inhibitor group and the DVT and GRP94 inhibitor group as compared to the DVT group (both *p* < 0.01, Figure 3A,B).

We further examined whether PDI and GRP94 regulate GPIIb or GPIIIa subset expression and activity. CD61 platelets detected by FCM assays show activated GPIIIa expression, CD41 platelets show activated GPIIb expression, and CD62p platelets show activated platelets. GPIIIa platelets are increased in the DVT group compared with the sham group (*p* < 0.05, Figure 3C,D). GPIIIa^down^CD62p platelets are significantly increased in the DVT and PDI inhibitor group compared with the DVT group (*p* < 0.001, Figure 3C,E). The platelet activation conclusions of the FCM in vitro experiment are similar to the experiment in vivo. EVs are secreted by hypoxia EVs (H-EVs) or normal EVs (N-EVs) HUVECs and it are co-cultured with platelets; the PDI inhibitor and the GRP94 inhibitor were used to observe the activation of platelets. We find that the CD62p expression on the total platelet surface is significantly increased in the H-EVs group as compared to the N-EVs group, and we also find that PDI inhibitor or GRP94 inhibitor decrease the CD62p expression induced by hypoxic conditions (both *p* < 0.001, Figure 3F,G). Similar results confirm that GPIIb/IIIa platelets are significantly downregulated in the H-EVs and PDI inhibitor group and the H-EVs and GRP94 inhibitor group compared with the H-EVs group (both *p* < 0.001, Figure 3F,G). The proportion of GPIIb/IIIa and CD62p platelets is significantly increased in the H-EVs group compared with the N-EVs group, but the PDI inhibitor and the GRP94 inhibitor decrease GPIIb/IIIa and CD62p platelets (both *p* < 0.001, Figure 3F,G). In summary, PDI and GRP94 negatively regulate the GPIIb submit, the GPIIIa submit, and the GPIIb/IIIa integrin expression when platelets are activated.

### 3.4. Hypoxia-Induced Endothelial-Derived EVs Carrying PDI Endocytosed by Platelets Induced GRP94 Monomers to Change into Dimers, Resulting in Platelet Activation

To further address the mechanism by which PDI and GRP94 regulate GPIIb/IIIa expression, IF was performed to detect the expression of GPIIb and GRP94 on thrombi. Significantly less GRP94 immunostaining in thrombi is observed in the DVT and PDI inhibitor group and the DVT and GRP94 inhibitor group (*p* < 0.001, Supplementary Figure S5H,I) than in the DVT group. The result indicates that PDI significantly increases GRP94 expression.

**Figure 4:**
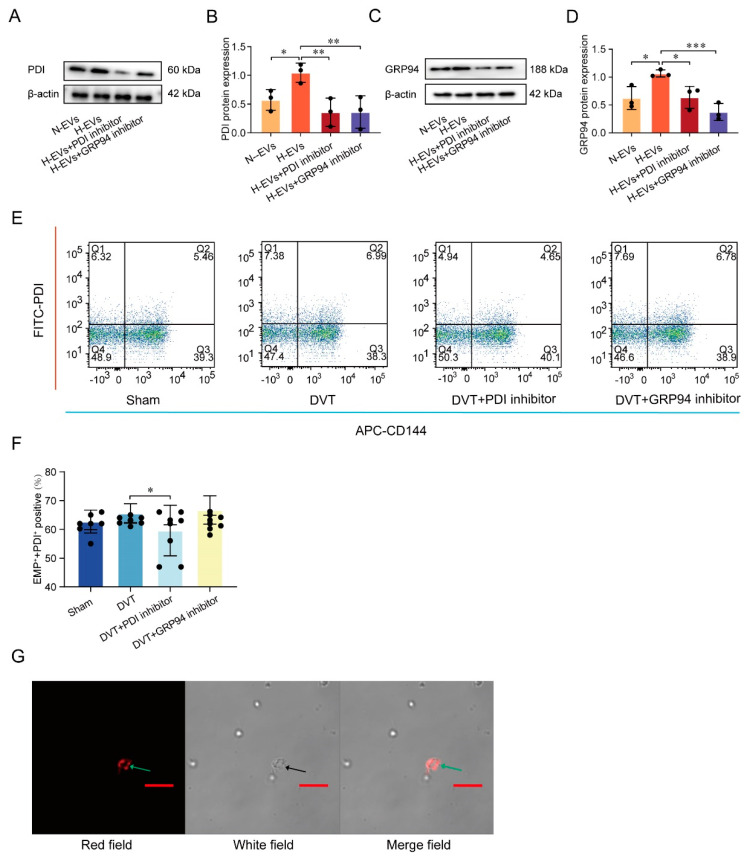
Hypoxia-induced endothelial-derived EVs carrying PDI endocytosed by platelets induced the GRP94 monomer into a dimer, resulting in platelet activation. (**A**) Representative Western blot bands of PDI. (**B**) Quantification of PDI. (**C**) Representative native PAGE bands of the GRP94 dimer. (**D**) Quantification of the GRP94 dimer. (**E**) Detection the PDI expression on CD144-positive EVs in peripheral blood of mice by FCM. (**F**) Statistical analysis of endothelial-derived EVs carrying PDI expression in the plasma of mice. (**G**) Representative microscope images displaying the EVs uptake assay, which was conducted to confirm the uptake of PKH26-labeled EVs (red fluorescence, indicated by green arrow) into the recipient platelet (indicated by black arrow); the PKH26 experiment only elaborates on the phenomenon in which endothelial-derived EVs can be endocytosed by platelets, and it is not a quantitative experiment, so there is no statistical graph. Scale bar for red line = 10 μm. EVs, extracellular vesicles; * *p* < 0.05, ** *p* < 0.01, *** *p* < 0.001. DVT, deep vein thrombosis; PDI, protein disulfide isomerase; GRP94, glucose-regulated protein 94.

Hypoxia or normal HUVECs were co-cultured with platelets in vivo. Western blot was performed in platelets to further explore the underlying mechanism involved in the regulation of the PDI–GRP94–GPIIb/IIIa pathway. Intriguingly, compared with the H-EVs group, the protein level of PDI is significantly downregulated in the N-EVs group (*p* < 0.05, Figure 4A,B) and the H-EVs and PDI inhibitor group (*p* < 0.01, Figure 4A,B). GRP94 is a monomer chain of 94 kDa in SDS-PAGE. A GRP94 dimer chain of 184 kDa is detected on native PAGE. The GRP94 dimer protein level is significantly higher in the H-EVs group compared with the N-EVs group (*p* < 0.05, Figure 4C,D). Compared with the H-EVs group, GRP94 dimer protein level is significantly lower in the H-EVs and PDI inhibitor group (*p* < 0.05, Figure 4C,D). These results show that PDI induces the GRP94 monomer to change into a dimer, which increases GPIIb/IIIa expression on the platelet surface. The above results suggest that platelets are activated by the PDI–GRP94–GPIIb/IIIa pathway, excluding endogenous PDI of platelet factors.

Under conditions of an injured inferior vena cava, the PDI content on endothelial cells is elevated, but whether the elevated PDI is endocytosed by platelets remains unknown. Vascular endothelial cadherin, also known as CD144, is a specific marker for endothelial cells. Therefore, CD144-positive endothelial-derived EVs are considered to be endothelial-derived EVs. FCM was used to detect the PDI expression on CD144-positive EVs in the plasma of mice. Compared with the sham group, PDI expression on CD144-positive EVs is significantly higher in the DVT group. PDI expression on CD144-positive EVs is significantly lower in the DVT and PDI inhibitor group compared with the DVT group (*p* < 0.05, Figure 4E,F), while no significant downregulation is seen in the DVT and GRP94 inhibitor group (*p* > 0.05, Figure 4E,F). This indicates that the PDI inhibitor, but not the GRP94 inhibitor, suppresses PDI expression on EVs. Furthermore, we confirm whether endothelial-derived EVs can be endocytosed by other cells of the cellular microenvironment. PKH26-labeled endothelial-derived EVs were co-incubated with platelets for 20 min. The results show that red, scattered fluorescent spots are visible within the platelets, which are PKH26-labeled endothelial-derived EVs (Figure 4G). The PKH26 experiment only elaborates on the phenomenon in which endothelial-derived EVs can be endocytosed by platelets, and it is not a quantitative experiment, so there is no statistical graph. These results reveal that endothelial-derived EVs are endocytosed into the platelets and that endothelial-derived EVs carrying PDI increase platelet activation though the PDI–GRP94–GPIIb/IIIa pathway.

### 3.5. PDI Regulates Platelet Function and Hypoxia-Induced Endothelial-Derived EVs Induce GPIIb/IIIa Conformation Activation

**Figure 5:**
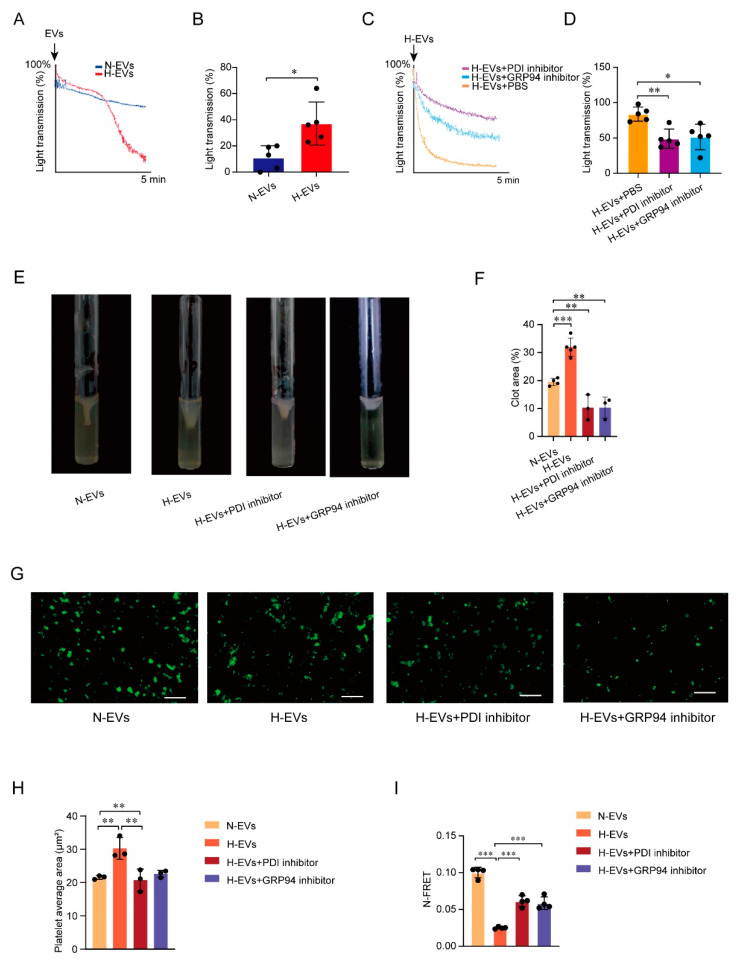
PDI regulates platelet function and hypoxia induces EV-induced GPIIb/IIIa conformation activation. (**A**) Representative images display H-EVs-induced platelet aggregation curves. (**B**) H-EVs-induced platelet aggregation in platelets. (**C**) Representative images of ADP-induced platelet aggregation curves. (**D**) ADP-induced platelet aggregation in platelets. (**E**) Clot retraction of washed platelets was initiated by adding fibrinogen and thrombin. Representative images display clot retraction. (**F**) Quantification of the average retraction area. (**G**) Platelet spreading was visualized under an epifluorescence microscope. Scale bar = 20 μm. (**H**) Quantification of the average clot size of an individual platelet. (**I**) The 293T cells were transfected with GPIIb and GPIIIa overexpression fluorescent plasmids, and FRET was used to detect the conformational changes in platelet GPIIb/IIIa. Statistical analysis of the energy transfer efficiency of platelet GPIIb/IIIa. * *p* < 0.05, ** *p* < 0.01, *** *p* < 0.001. PBS, phosphate buffer saline; EVs, extracellular vesicles; DVT, deep vein thrombosis; PDI, protein disulfide isomerase; GRP94, glucose = regulated protein 94.

The exact role of PDI in platelet function and DVT formation remains poorly understood. In this study, we characterized the role of PDI in platelet activation and function, including platelet aggregation, activation, and molecular conformation, and platelet spreading, as well as in clot retraction. A platelet aggregation experiment was employed to check the effect of EVs on platelets in a hypoxic environment. H-EVs from the HUVECs significantly stimulate platelet aggregation compared with the N-EVs group (*p* < 0.05, Figure 5A,B). Moreover, if platelet plasma is preincubated with PDI or GRP94 inhibitors for 10 min, ADP-induced platelet aggregation is partly inhibited in the presence of the PDI inhibitor and the GRP94 inhibitor. Platelet aggregation is significantly inhibited in the H-EVs and PDI inhibitor group (*p* < 0.01, Figure 5C,D) and the H-EVs and GRP94 inhibitor group (*p* < 0.05, Figure 5C,D) compared with the H-EVs and PBS group, indicating a positive correlation between PDI or GRP94 and platelet aggregation. Further exploring the mechanism underlying platelet aggregation, we found that the PDI inhibitor or the GRP94 inhibitor may affect aggregation occurrence by inhibiting the expression of GPIIb/IIIa on platelets (Figure 3C–G).

Platelets clot retraction exhibits a significant reduction in the DVT and PDI inhibitor group and the DVT and GRP94 inhibitor group compared to the DVT group (Figure 5E,F).

In the H-EVs and PDI inhibitor group and the H-EVs and GRP94 inhibitor group, we find significantly impaired platelet spreading on fibrinogen as compared to the H-EVs group (Figure 5G,H). We also find that platelet spreading is significantly impaired in the H-EVs group compared with N-EVs group (Figure 5G,H). These results confirm that endothelial-derived EVs, PDI, and GRP94 all play a role in platelet spreading.

PDI has been demonstrated to increase expression levels of GPIIb/IIIa. However, whether PDI, as a disulfide isomerase, could increase GPIIb/IIIa activity warrants further investigation. The 293T cells were transfected with GFP–GPIIb and mCherry–GPIIIa overexpression fluorescent plasmids, and FRET was used to detect changes in platelet GPIIb/IIIa conformation. Hypoxia-induced EVs from HUVECs were incubated with 293T after transfection for 2 h. The N-FRET value is significantly reduced in the H-EVs group compared with the N-EVs group, in which the phenomenon is rescued by the PDI inhibitor and the GRP94 inhibitor (both *p* < 0.001, Figure 5I), indicating that PDI affects the spatial conformational change in GPIIb/IIIa. Together, these results demonstrate that PDI plays a fundamental role in mediating platelet function and GPIIb/IIIa conformational change.

## 4. Discussion

**Figure 6:**
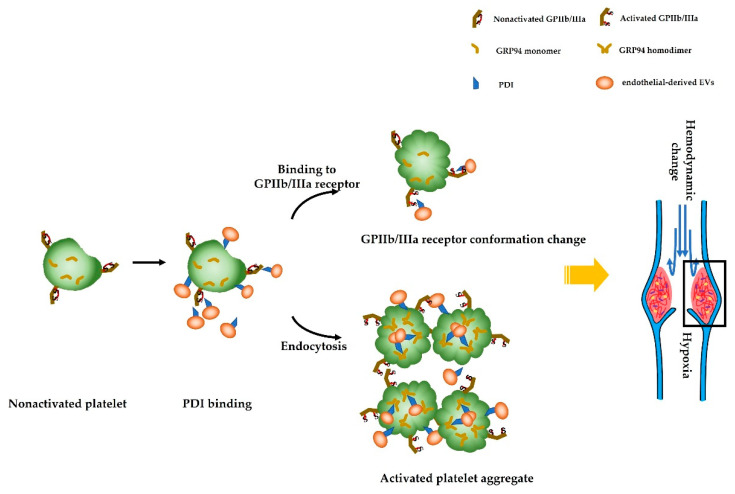
Schematic picture of endothelial-derived EVs as a source of platelet activation in DVT. On the one hand, PDI carried by endothelial-derived EVs recognize GPIIb/IIIa receptors in platelets, which results in platelet activation and aggregation. On the other hand, PDI endocytosed by platelets cause GRP94 monomers to change to homodimers, which further triggers platelet activation and contributes to deep vein thrombus development.

In this study, it is confirmed that platelet activation and endothelial-derived EVs carrying PDI are significantly increased in DVT mice. The elevated endothelial-derived EVs could activate platelets by allosteric GPIIb/IIIa receptors through PDI on the surface. Meanwhile, platelets could endocytose PDI-containing endothelial-derived EVs, which induces the dimerization of monomeric GRP94, and the expression of platelet surface GPIIb/IIIa content is increased as a result (Figure 6). In this context, patients at risk of DVT might benefit from antiplatelet PDI therapy as primary prevention.

We show that a PDI inhibitor, CCF642, could act against DVT in vivo and in vitro. The importance of PDI in the early stages thrombosis has been growingly appreciated, and PDI inhibitors are being regarded as potential new anti-thrombosis medications. Rutin, one of the most potent PDI inhibitors, was reported to suppress platelet aggregation and thrombosis in animal models, but further studies and clinical translation were restricted due to its low aqueous solubility and oral bioavailability [22]. Previous research demonstrated that CCF642 treatment reduced body weight loss and muscle wasting in vivo and blocked PDI-derived EVs of myoblast apoptosis in vitro [23]. Kamarehei et al. investigated the capacity of CCF642 in reducing the expression of ER stress indicators and neuroinflammation in the brain, by using an experimental autoimmune encephalomyelitis mouse model [24]. In our study, by using a DVT mouse model, we discovered that CCF642 could inhibit platelet activation. A similar antithrombotic efficacy is found by comparing with rivaroxaban, which reduces platelet activation, inhibits the activation of extrinsic coagulation cascade, and reduces the formation of thrombi [25]. Additionally, suppression of PDI causes a sharp increase in misfolded poly-ubiquitinated proteins and ER stress, including ERp46, ERp57, and PDI [26,27]. It is encouraging that CCF642 could specifically inhibit PDI, while it will not lead to endoplasmic reticulum stress after treatment.

Platelet involvement in arterial thromboembolism and an association with adverse cardiovascular events has been reported; however, its function in DVT remains unclear. It has been suggested that platelet activation plays a role in the pathogenesis of VTE, as elevated levels of platelet-derived EVs are linked to an increased chance of future incidents of VTE [4]. In our study, we find that the percentage of activated platelets in mice with DVT is significantly higher than in the control group, demonstrating the crucial role of platelets in this condition. Nonetheless, many recent studies show that endothelial-derived EVs play a key role in hemostasis and thrombosis, and that they are involved in various thromboembolic events, including thalassemia [28], atrial fibrillation [16], venous thrombosis [29], and other diseases. Numerous studies demonstrate that as endothelial dysfunction increases, more endothelial-derived EVs are released [30], which is consistent with our results. Additionally, we discovered that endothelial-derived EVs can be endocytosed by platelets using PKH26. Endothelial-derived EVs might, therefore, be a significant cause of platelet activation in DVT.

The mechanism of platelet activation initiation caused by endothelial-derived EVs was further investigated. PDI is found on endothelial-derived EVs [31], and platelet surface-bound PDI has been demonstrated to be of vital importance to GPIIb/IIIa integrin activation and artery thrombosis [32]. However, the role of endothelial-derived EVs carrying PDI has remained speculative with regard to venous thrombosis. Our present study shows that the levels of endothelial-derived EVs carrying PDI are significantly elevated in the plasma of DVT mice, and GPIIb/IIIa on the surface of activated platelets or thrombi is significantly increased, whereas PDI inhibition results in a reduction in the activation levels of endothelial-derived EVs carrying PDI and GPIIb/IIIa. The study also finds conformational change in GPIIb/IIIa after platelets are stimulated by endothelial-derived EVs carrying PDI. Thus, it is believed that endothelial-derived EVs carrying PDI in the plasma play an important role in platelet activation, leading to DVT.

The formation of thrombus requires PDI at every stage. Our results in platelets are analogous to previous reports, in which parallel expression of PDI and GRP94 is shown in B cells [13]. In addition, the role of GRP94 dimers in their biological activity for platelet activation was investigated. Studies dealing with the role of GRP94 in platelet integrin reactivity have been reported in recent years. Jackson et al. [11] found that Kung 65, a GRP94 inhibitor, is associated with lower GPIIb/IIIa expression on platelet surfaces. In our study, we show that the expression of GPIIb/IIIa on the platelet surface is reduced when GRP94 dimerization is inhibited. Therefore, we discover, for the first time, that PDI plays a critical role in the formation of disulfide bonds within dimeric GRP94, and participates in DVT by directly enhancing platelet activation.

Disulfide bonds play a pivotal role in maintaining the natural structures of proteins to ensure the performance of their normal biological functions. The formation of disulfide bonds is a key rate-limiting step in protein folding, and the efficiency of spontaneous disulfide bond formation is far lower than that under enzymatic catalysis [33]. In a variety of cardiovascular diseases, PDI, a prototypical thiol isomerase, catalyzes the creation of thiol–disulfide bonds during protein folding [34]. While thiol isomerases do not activate GPIIb/IIIa by themselves, they are required for transformation to the high affinity state [35]. Nevertheless, it is unclear whether PDI affects the disulfide bonds of GPIIb/IIIa in platelets during DVT. It is reported that PDI could regulate structural changes on the surface of platelet GPIIb/IIIa in diabetes [8]. For the first time, we put forward FRET technology to determine the role of PDI in converting GPIIb/IIIa from its resting to its active conformation. Furthermore, the biological activity of GRP94 requires the maintenance of its dimeric configuration, which seem to be stabilized by disulfide bonds [36]. In short, as endothelial-derived EVs carrying PDI are endocytosed by platelets, the platelet activation is mediated by allosteric GPIIb/IIIa receptors on its surface and dimeric GRP94 inside.

## 5. Limitations

Further research is required on the combined effects of anticoagulation treatment and PDI–GRP94–GPIIb/IIIa pathway inhibition, as well as the relationship between platelet activation of GPIIb/IIIa and anticoagulation therapy. Moreover, we intend to perform some research on the use of antiplatelet agents in spite of anticoagulants based upon the present results. Finally, additional clinical trials in DVT patients to determine the effect of the PDI inhibitor and the GRP94 inhibitor on bioavailability, rapid degradation, and gastrointestinal absorption are needed. In fact, we did not conduct clinical studies regarding the timing of the testing of elevated endothelial-derived EVs in the diagnostic algorithm of DVT and do not have clinical data, but it is suggested that, combined with the venous thromboembolism score of nursing care, further studies can measure endothelium-derived extracellular vesicles in high-risk DVT patients and conduct follow-up studies.

## 6. Conclusions

Platelet activation is considerably higher in DVT mice with increased PDI on endothelial-derived EVs. The elevated endothelial-derived EVs tend to activate platelets via allosteric GPIIb/IIIa receptors through PDI on the surface of endothelial-derived EVs. Endothelial-derived EVs carrying PDI endocytosed by platelets promote the change of the GRP94 monomer into a dimer, thus, increasing the GPIIb/IIIa content. The current study provides evidence that the PDI–GRP94–GPIIb/IIIa pathway contributes to DVT, and sheds light on a therapeutic approach to prevent DVT. Our study implies that endothelial-derived extracellular vesicles could chelate the known PDI inhibitor troxerutin as a complex to promote extracellular vesicles’ antithrombotic properties.

## Figures and Tables

**Table 1:** The length of thrombi.

Group Name	Length of Thrombi (mm)	*p*
DVT	3.7 ± 3.6	
DVT and PDI inhibitor	0.8 ± 1.2	0.009
DVT and GRP94 inhibitor	1.4 ± 1.8	0.035
DVT and rivaroxaban	2.5 ± 2.1	0.295

Abbreviations: DVT, deep vein thrombosis; PDI, protein disulfide isomerase; GRP94, glucose-regulated protein 94. *p* = 0.036 (DVT and GRP94 inhibitor vs. DVT), *p* = 0.007 (DVT and PDI inhibitor vs. DVT).

## Data Availability

Data, materials, and software information supporting the conclusions of this article are included within the article and its additional file.

## Supplementary Materials

The following supporting information can be downloaded at: https://www.mdpi.com/article/10.3390/jcm12134265/s1, Figure S1: Identification of HUVECs; Figure S2: Migration assay of HUVECs; Figure S3: Identification of epithelial cell-derived EVs; Figure S4: A DVT mouse model establishment and GRP94 immunostaining for thrombus surface; Figure S5: PDI inhibitor and GRP94 inhibitor drug toxicity and platelet activation levels in DVT mouse were detected. Table S1: primer information.

## Abbreviations

DVT (deep vein thrombosis), EVs (extracellular vesicles), N-EVs (normal extracellular vesicles), H-EVs (hypoxia extracellular vesicles), PDI (protein disulfide-isomerase), GRP94 (glucose regulatory protein 94), PRP (platelet-rich plasma), PPP (platelet-poor plasma), FCM (flow cytometry), HUVECs (human umbilical vein endothelial cells), FRET (fluorescence resonance energy transfer), ADP (adenosine 5′-diphosphate), PBS (phosphate buffer saline).
