# Supplementary material for: Deep Vein Thrombosis Is Facilitated by Endothelial-Derived Extracellular Vesicles via the PDI–GRP94–GPIIb/IIIa Pathway in Mice
Source: J Clin Med. 2023 Jun 26;12(13):4265. doi: 10.3390/jcm12134265 (PMC10343006; doi:10.3390/jcm12134265)
Supplement: Supplementary file 1 [file jcm-12-04265-s001.zip › jcm-2293505-supplementary.pdf]

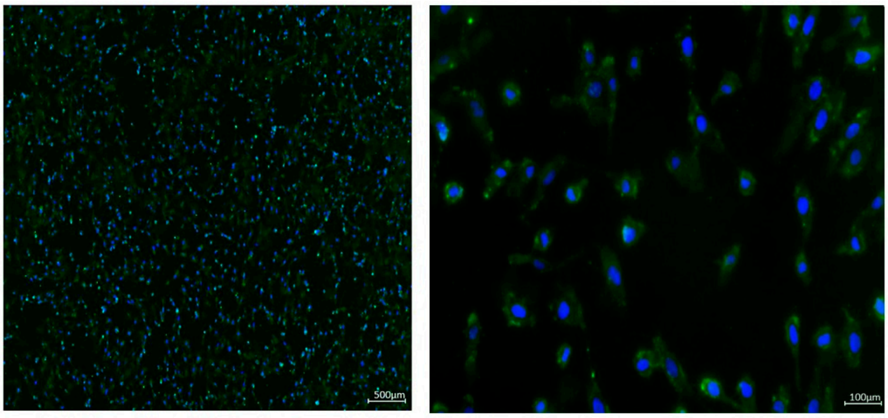

IF

Magnification, ×40 | Magnification, ×200

Supplementary Figure S1. Identification of HUVECs. CD31 IF staining was performed on HUVECs, which was subjected to Inverted fluorescence microscope. IF, immunofluorescent. HUVECs, human umbilical vein endothelial cells.

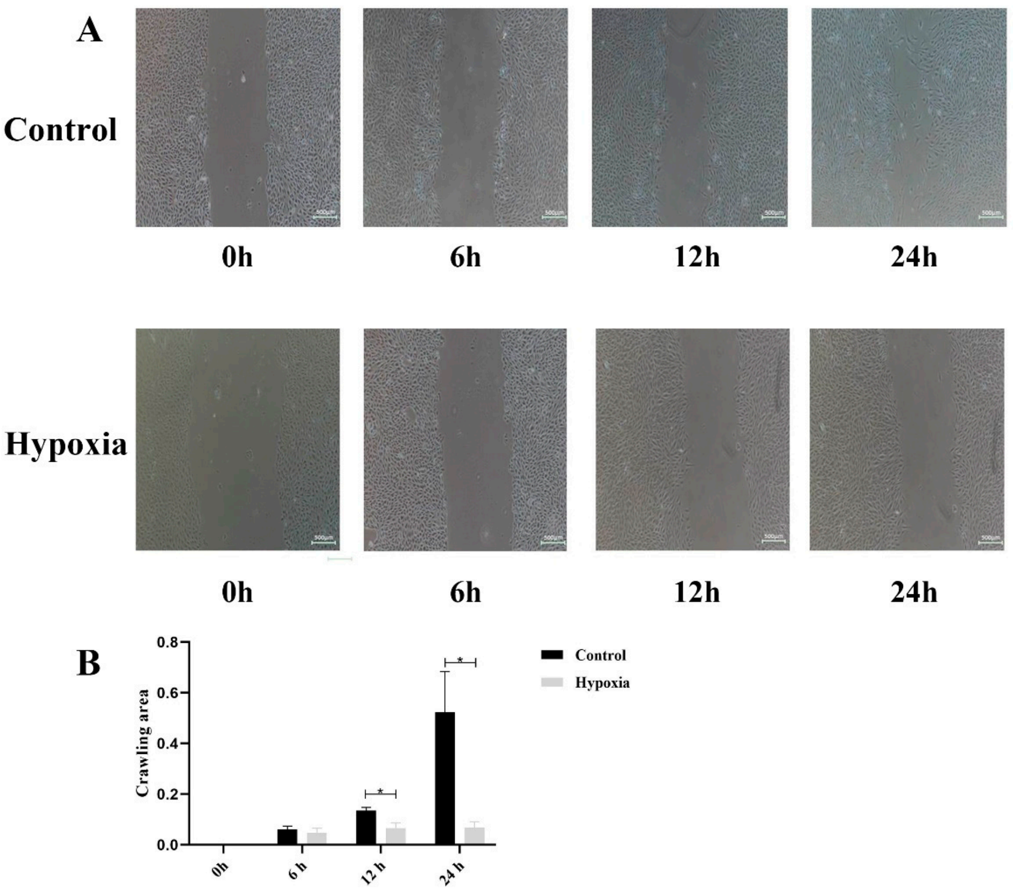

Supplementary Figure S2. Migration assay of HUVECs. **A** A scratch was created in the cell monolayer, the crawling area was captured 0 hours, 6 hours, 12 hours, and 24 hours after the scratch was made. **B** The crawling area was compared between the control group and the hypoxia group at consecutive time points.

* $p < 0.05$. HUVECs, human umbilical vein endothelial cells.

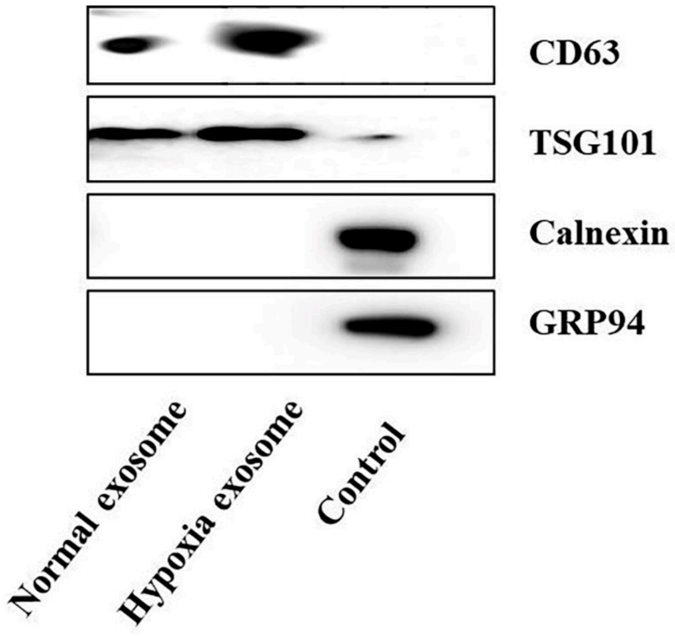

Supplementary Figure S3. Identification of epithelial cell-derived EVs. CD63 and TSG101 are positive indicators for exosome; Calnexin and GRP94 are negative indicators for exosomes. EVs, extracellular vesicles.

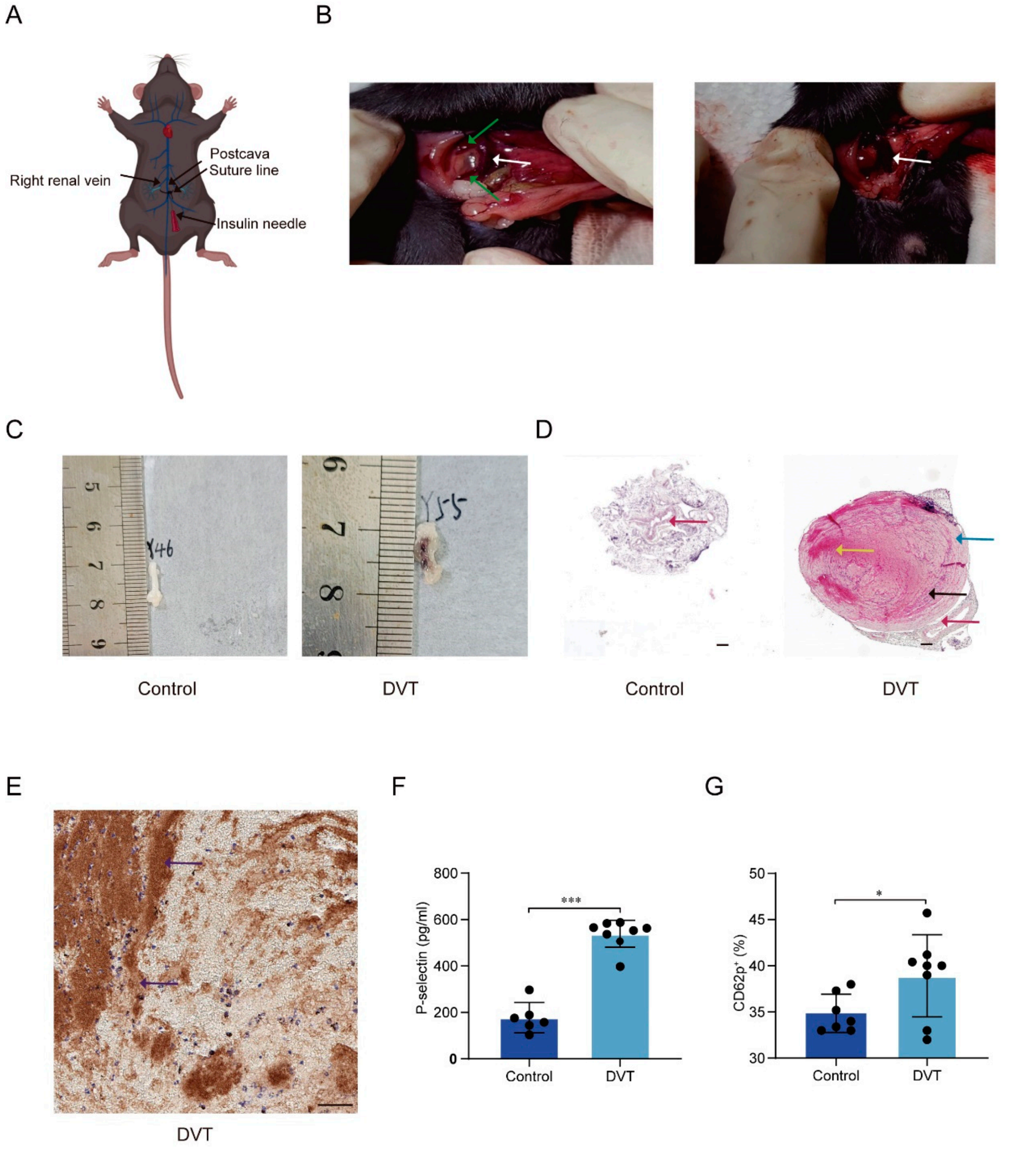

Supplementary Figure S4. A DVT mouse model establishment and GRP94 immunostaining for thrombus surface. **A.** Scheme of DVT operation. **B.** Right renal vein (indicated by green arrow), inferior vena cava (indicated by write arrow), and branches. After ligation of inferior vena cava and branches the inferior vena cava is filled. **C.** Morphological thrombus comparison between control group and DVT group. In DVT group, the thrombus has adhered to the vascular wall, the color of thrombus was black and textural hardness is hard than control group. **D.** HE staining of thrombus was not anything was found in inferior vena cave (indicated by pink arrow) of control group, but in DVT group, it is firmly attached to inferior vena cave (indicated by pink arrow), white blood cells in DVT (indicated by black arrow), red blood cells have no nucleus (indicated by yellow arrow), platelets and fibrinogen (indicated by blue arrow). Scale bar = 100 μm. **E.** Platelet was detected by IHC (indicated by purple arrow). Scale bar = 50 μm. **F.** Detection of

plasma P-selectin in mice by ELISA. **G.** Detection of CD62p on platelet surface in mice by FCM. *$P < 0.05$, ***$P < 0.001$.

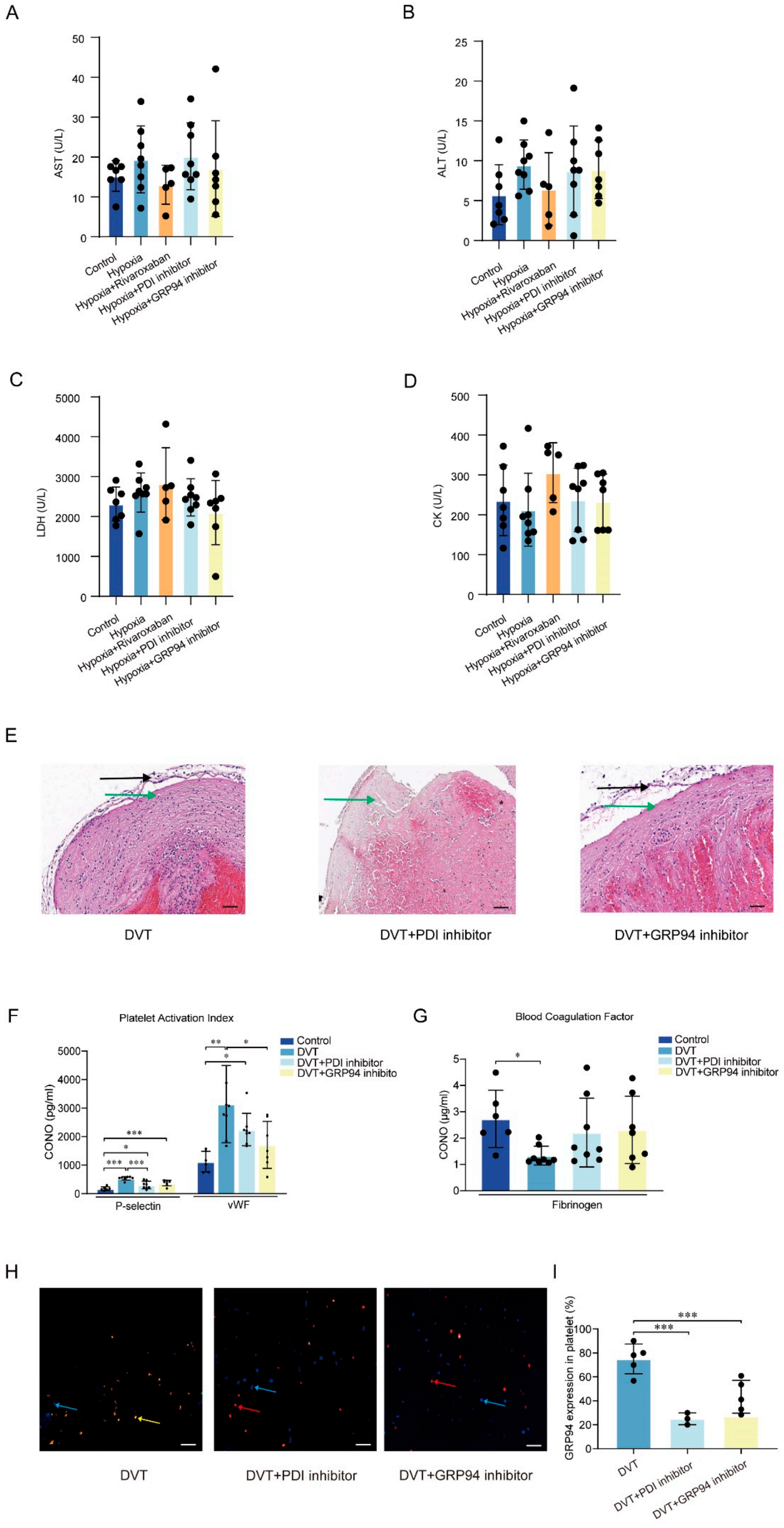

A
AST (U/L)
Control
Hypoxia
Hypoxia+Rivaroxaban
Hypoxia+PDI inhibitor
Hypoxia+GRP94 inhibitor
B
ALT (U/L)
C
LDH (U/L)
D
CK (U/L)
E
DVT
DVT+PDI inhibitor
DVT+GRP94 inhibitor
F
Platelet Activation Index
CONO (pg/ml)
P-selectin
vWF
Control
DVT
DVT+PDI inhibitor
DVT+GRP94 inhibito
G
Blood Coagulation Factor
CONO (μg/ml)
Fibrinogen
Control
DVT
DVT+PDI inhibitor
DVT+GRP94 inhibitor
H
DVT
DVT+PDI inhibitor
DVT+GRP94 inhibitor
I
GRP94 expression in platelet (%)
DVT
DVT+PDI inhibitor
DVT+GRP94 inhibitor

Supplementary Figure S5. PDI inhibitor and GRP94 inhibitor drug toxicity and platelet activation levels in DVT mouse were detected. **(A and B)** AST and ALT level were no significant change between the groups (*P* > 0.05). **(C and D)** LDH and CK level were no significant change between the groups (*P* > 0.05). **E.** Representative microscope image display of the HE staining of a thrombus (indicated by green arrow) and the vessel wall (indicated by black arrow). Scale bar = 20 μm. **F.** Detection of plasma P-selectin and vWF in mice by ELISA. **G.** Detection of plasma fibrinogen in mice by ELISA. **H**. Representative fluorescent microscope images display thrombus of mice by IF staining of GRP94 (green fluorescence) and GPIIb/IIIa-labeled platelets (red fluorescence, indicated by red arrow), the color of fluorescence (indicated by yellow arrow) is orange after merging green and red fluorescence. The sections were also stained with DAPI to show nuclei (blue fluorescence, indicated by a blue arrow). Scale bar = 20 μm. **I.** Quantitative analyses of GRP94 expression in GPIIb/IIIa-labeled platelets on thrombus. *$P < 0.05$, **$P < 0.01$, ***$P < 0.001$.

Supplementary Table S1: primer information

Primers ID	**Seq (5′→3′)**
Forward primer-ITGA2B (70711-1)	GTTTAAACGGGCCCTCTAGACGCCACCATGGCCAGAGCTTTGTGTCCACTGCAAG
Reverse primer- ITGA2B (70711-1)	CAGCGGTTTAACTATCTAGATCACTCCCCCTCTTCATCATCTTCTTC
Forward primer-ITGB3 (70708-1)	TTGGTACCGAGCTCGGATCCCGCCACCATGCGAGCGCGGCCGCGGCCCCGGCCG
Reverse primer-ITGB3 (70708-1)	ACGGGCCCTCTAGACTCGAGTTAAGTGCCCCGGTACGTGATATTGGTG

ITGB3 sequencing:

TAACTAGAGAACCCACTGCTTACTGGCTTATCGAAATTAATACGACTCACTATAGGGAGACCCAAGCTGGCTAGCGTTTAAACTTAAGCTTGGTACCGAGCTCGGATCCCGCCACCATGCGAGCGCGGCCGCGGCCCCGGCCGCTCTGGGCGACTGTGCTGGCGCTGGGGGCGCTGGCGGGCGTTGGCGTAGGAATGGTGAGCAAGGGCGAGGAGGATAACATGGCCATCATCAAGGAGTTCATGCGCTTCAAG

GTGCACATGGAGGGCTCCGTGAACGGCCACGAGTTCGAGATCGAGGGCGAGGGCGAGGGCCG

CCCCTACGAGGGCACCCAGACCGCCAAGCTGAAGGTGACCAAGGGTGGCCCCCTGCCCTTCGC

CTGGGACATCCTGTCCCCTCAGTTCATGTACGGCTCCAAGGCCTACGTGAAGCACCCCGCCGAC

ATCCCCGACTACTTGAAGCTGTCCTTCCCCGAGGGCTTCAAGTGGGAGCGCGTGATGAACTTCG

AGGACGGCGGCGTGGTGACCGTGACCCAGGACTCCTCCCTGCAGGACGGCGAGTTCATCTACA

AGGTGAAGCTGCGCGGCACCAACTTCCCCTCCGACGGCCCCGTAATGCAGAAGAAGACCATGG

GCTGGGAGGCCTCCTCCGAGCGGATGTACCCCGAGGACGGCGCCCTGAAGGGCGAGATCAAGC

AGAGGCTGAAGCTGAAGGACGGCGGCCACTACGACGCTGAGGTCAAGACCACCTACAAGGCC

AAGAAGCCCGTGCAGCTGCCCGGCGCCTACAACGTCAACATCAAGTTGGACATCACCTCCCAC

AACGAGGACTACACCATCGTGGAACAGTACGAACGCGCCGAGGGCCGCCACTCCACCGGCGG

CATGGACGAGCTGTACAAGGGAGGTGGAGGATCAGGGGGTGGGGGATCCGGCGGTGGCGGAT

CTGGGCCCAACATCTGTACCACGCGAGGTGTGAGCTCCTGCCAGCAGTGCCTGGCTGTGAGCCC

CATGTGTGCCTGGTGCTCTGATGAGGCCCTGCCTCTGGGCTCACCTCGCTGTGACCTGAAGGAG

AATCTGCTGAAGGATAACTGTGCCCCAGAATCCATCGAGTTCCCAGTGAGTGAGGCCCGAGTA

CTAGAGGACAGGCCCCTCAGCGACAAGGGCTCTGGAGACAGCTCCCAGGTCACTCAAGTCAGT

CCCCAGAGGATTGCACTCCGGCTCCGGCCAGATGATTCGAAGAATTTCTCCATCCAAGTGCGGC

AGGTGGAGGATTACCCTGTGGACATCTACTACTTGATGGACCTGTCTTACTCCATGAAGGATGA

TCTGTGGAGCATCCAGAACCTGGGTACCAAGCTGGCCACCCAGATGCGAAAGCTCACCAGTAA

CCTGCGGATTGGCTTCGGGGCATTTGTGGACAAGCCTGTGTCACCATACATGTATATCTCCCCAC

CAGAGGCCCTCGAAAACCCCTGCTATGATATGAAGACCACCTGCTTGCCCATGTTTGGCTACAA

ACACGTGCTGACGCTAACTGACCAGGTGACCCGCTTCAATGAGGAAGTGAAGAAGCAGAGTGT

GTCACGGAACCGAGATGCCCCAGAGGGTGGCTTTGATGCCATCATGCAGGCTACAGTCTGTGAT

GAAAAGATTGGCTGGAGGAATGATGCATCCCACTTGCTGGTGTTTACCACTGATGCCAAGACTC

ATATAGCATTGGACGGAAGGCTGGCAGGCATTGTCCAGCCTAATGACGGGCAGTGTCATGTTG

GTAGTGACAATCATTACTCTGCCTCCACTACCATGGATTATCCCTCTTTGGGGCTGATGACTGAG

AAGCTATCCCAGAAAAACATCAATTTGATCTTTGCAGTGACTGAAAATGTAGTCAATCTCTATC

AGAACTATAGTGAGCTCATCCCAGGGACCACAGTTGGGGTTCTGTCCATGGATTCCAGCAATGT

CCTCCAGCTCATTGTTGATGCTTATGGGAAAATCCGTTCTAAAGTAGAGCTGGAAGTGCGTGAC

CTCCCTGAAGAGTTGTCTCTATCCTTCAATGCCACCTGCCTCAACAATGAGGTCATCCCTGGCCT

CAAGTCTTGTATGGGACTCAAGATTGGAGACACGGTGAGCTTCAGCATTGAGGCCAAGGTGCG

AGGCTGTCCCCAGGAGAAGGAGAAGTCCTTTACCATAAAGCCCGTGGGCTTCAAGGACAGCCT

GATCGTCCAGGTCACCTTTGATTGTGACTGTGCCTGCCAGGCCCAAGCTGAACCTAATAGCCAT

CGCTGCAACAATGGCAATGGGACCTTTGAGTGTGGGGTATGCCGTTGTGGGCCTGGCTGGCTGG

GATCCCAGTGTGAGTGCTCAGAGGAGGACTATCGCCCTTCCCAGCAGGACGAATGCAGCCCCC

GGGAGGGTCAGCCCGTCTGCAGCCAGCGGGGCGAGTGCCTCTGTGGTCAATGTGTCTGCCACA

GCAGTGACTTTGGCAAGATCACGGGCAAGTACTGCGAGTGTGACGACTTCTCCTGTGTCCGCTA

CAAGGGGGAGATGTGCTCAGGCCATGGCCAGTGCAGCTGTGGGGACTGCCTGTGTGACTCCGA

CTGGACCGGCTACTACTGCAACTGTACCACGCGTACTGACACCTGCATGTCCAGCAATGGGCTG

CTGTGCAGCGGCCGCGGCAAGTGTGAATGTGGCAGCTGTGTCTGTATCCAGCCGGGCTCCTATG

GGGACACCTGTGAGAAGTGCCCCACCTGCCCAGATGCCTGCACCTTTAAGAAAGAATGTGTGG

AGTGTAAGAAGTTTGACCGGGGAGCCCTACATGACGAAAATACCTGCAACCGTTACTGCCGTG

ACGAGATTGAGTCAGTGAAAGAGCTTAAGGACACTGGCAAGGATGCAGTGAATTGTACCTATA

AGAATGAGGATGACTGTGTCGTCAGATTCCAGTACTATGAAGATTCTAGTGGAAAGTCCATCCT

GTATGTGGTAGAAGAGCCAGAGTGTCCCAAGGGCCCTGACATCCTGGTGGTCCTGCTCTCAGTG

ATGGGGGCCATTCTGCTCATTGGCCTTGCCGCCCTGCTCATCTGGAAACTCCTCATCACCATCCA

CGACCGAAAAGAATTCGCTAAATTTGAGGAAGAACGCGCCAGAGCAAAATGGGACACAGCCA

ACAACCCACTGTATAAAGAGGCCACGTCTACCTTCACCAATATCACGTACCGGGGCACTTAACT

CGAGTCTAGAGGGCCCGTTTAAACCCGCTGATCAGCCTCGACTGTGCCTTCTAGTTGCCAGCCA

TCTGTTGTTTGCCCCTCCCCCGTGCCTTCCTTGACCCTGGAAGGTGCCACTCCCACTGTCCTTTCC

TAATAAAATGAGGAAATTGCATCGCATTGTCTGAGTAGGTGTCATTCTATTCTGGGGGGTGGGG

TGGGGCAGGACAGCAAGGGGGAGGATTGGGAAGACAATAGCAGGC

ITGA2B sequencing:

TTTGTTAGACGAAGCTTGGGCTGCAGGTCGACTCTAGAGGATCCCCGGGTACCGGTCGCCACCA

TGGCGGAGCCGAGCGGCTCGCCCGTGCACGTCCAGCTTCCCCAGCAGGCGGCCCCGGTGACAG

CGGCGGCGGCGGCGGCCCCGGCGGCCGCGACAGCAGCGCCGGCCCCGGCAGCTCCCGCGGCC

CCGGCCCCGGCCCCGGCCCCGGCGGCACAGGCTGTCGGCTGGCCCATCTGCAGGGACGCGTAC

GAGCTGCAGGAGGTTATCGGCAGTGGAGCTACTGCTGTGGTTCAGGCAGCCCTATGCAAACCC

AGGCAAGAACGTGTAGCAATAAAACGGATCAACTTGGAAAAATGCCAGACCAGTATGGATGA

ACTATTAAAAGAAATTCAAGCCATGAGTCAGTGCAGCCATCCCAACGTAGTGACCTATTACAC

CTCTTTTGTGGTCAAAGATGAACTTTGGCTGGTCATGAAATTACTAAGTGGAGGTTCAATGTTGG

ATATCATAAAATACATTGTCAACCGAGGAGAACACAAGAATGGAGTTCTGGAAGAGGCAATA

ATAGCAACAATTCTTAAAGAGGTTTTGGAAGGCTTAGACTATCTACACAGAAACGGTCAGATTC

ACAGGGATTTGAAAGCTGGTAATATTCTTCTGGGTGAGGATGGTTCAGTACAAATAGCAGATTT

TGGGGTAAGTGCGTTCCTAGCAACAGGGGGTGATGTTACCCGAAATAAAGTAAGAAAAACATT

CGTTGGCACCCCATGTTGGATGGCTCCTGAAGTCATGGAACAGGTGAGAGGCTATGACTTCAAG

GCTGACATGTGGAGTTTTGGAATAACTGCCATTGAATTAGCAACAGGAGCAGCGCCTTATCACA

AATATCCTCCCATGAAAGTGTTAATGTTGACTTTGCAAAATGATCCACCCACTTTGGAAACAGG

GGTAGAGGATAAAGAAATGATGAAAAAGTACGGCAAGTCCTTTAGAAAATTACTTTCACTGTG

TCTTCAGAAAGATCCTTCCAAAAGGCCCACAGCAGCAGAACTTTTAAAATGCAAATTCTTCCAG

AAAGCCAAGAACAGAGAGTACCTGATTGAGAAGCTGCTTACAAGAACACCAGACATAGCCCA

AAGAGCCAAAAAGGTAAGAAGAGTTCCTGGGTCAAGTGGTCACCTTCATAAAACCGAAGACG

GGGACTGGGAGTGGAGTGACGACGAGATGGATGAGAAGAGCGAAGAAGGGAAAGCAGCTTTT

TCTCAGGAAAAGTCACGAAGAGTAAAAGAAGAAAATCCAGAGATTGCAGTGAGTGCCAGCAC

CATCCCCGAACAAATACAGTCCCTCTCTGTGCACGACTCTCAGGGCCCACCCAATGCTAATGAA

GACTACAGAGAAGCTTCTTCTTGTGCCGTGAACCTCGTTTTGAGATTAAGAAACTCCAGAAAGG

AACTTAATGACATACGATTTGAGTTTACTCCAGGAAGAGATACAGCAGATGGTGTATCTCAGGA

GCTCTTCTCTGCTGGCTTGGTGGATGGTCACGATGTAGTTATAGTGGCTGCTAATTTACAGAAGA

TTGTAGATGATCCCAAAGCTTTAAAAACATTGACATTTAAGTTGGCTTCTGGCTGTGATGGGTCG

GAGATTCCTGATGAAGTGAAGCTGATTGGGTTTGCTCAGTTGAGTGTCAGCCCGGTCGCCACCA

T G G T G A G C A A G G G
